# Exploring Characteristics of Preoccupation and Failure to Adapt Among Patients Suffering From Adjustment Disorder: A Qualitative Study

**DOI:** 10.32872/cpe.11565

**Published:** 2024-06-28

**Authors:** Alexis Vancappel, Rania Chkili, David J. Eberle, Andreas Maercker, Wissam El-Hage, Rahel Bachem

**Affiliations:** 1 CHRU de Tours, Pôle de Psychiatrie-Addictologie, Centre Régional de Psychotraumatologie CVL, Tours, France; 2 Département de Psychologie, QualiPsy, Qualité de Vie et santé Psychologique, Université de Tours, Tours, France; 3 Department of Psychology, Universität Zürich, Zurich, Switzerland; 4 UMR 1253, iBrain, Université de Tours, Inserm, Tours, France; Philipps-University of Marburg, Marburg, Germany

**Keywords:** adjustment disorder, preoccupations, failure to adapt, ICD-11, coping strategies

## Abstract

**Background:**

Adjustment Disorder (AjD) is a frequent diagnosis in psychological and psychiatric consultations. Recently, the ICD-11 has introduced preoccupation and failure to adapt as core symptoms of AjD. However, empirical research that explores the various possible manifestations of preoccupation and failure to adapt in AjD patients is sparse. Therefore, the study aimed to explore patients’ experiences of the core symptoms of AjD in a qualitative study.

**Method:**

We recruited 16 patients suffering from ICD-11 AjD who filled in self-report questionnaires to assess sociodemographic information, adjustment disorder symptoms, anxiety and depression. Then, they participated in a semi-structured interview with a trained psychologist to explore the determinants and characteristics of their preoccupation and failure to adapt symptoms. Thematic analysis was applied to analyze the responses.

**Results:**

Six themes were identified in our analysis 1) Preoccupation triggers, 2) Preoccupations and negative emotions, 3) Strategies to stop preoccupation, 4) Consequences of preoccupation, 5) Manifestation of difficulties/failure to adapt and 6) Strategies to address difficulties/failure to adapt.

**Conclusion:**

We found partial congruence between our data and previous conceptualizations of AjD. Preoccupations seem to be time-consuming, center around stressors and their consequences, and be associated with negative emotions. Some preoccupations reported by the patients could also be labeled as ruminations or worries. The failure to adapt symptoms seemed to be broader than the exemplary symptoms highlighted in current measures of AjD.

## Highlights

- Qualitative data set out preoccupations and failure to adapt among patients suffering from AjD.
- The preocupations seem to overlap ruminations and worries.
- Preocupations are associated with negative emotions.

Adjustment disorder (AjD) is a maladaptive reaction to an identifiable psychosocial stressor or multiple critical life events (e.g. divorce, illness or disability, socio-economic problems, conflicts at home or work) that usually emerges within a month of the stressor. The ICD-11 has recently introduced a new conceptualization of AjD, focusing on the two core symptom clusters of preoccupation with the stressor or its consequences and failure to adapt symptoms (World Health Organization, 2019). These core symptoms must result in significant impairment of personal, social, educational, professional or other important areas of functioning. The definition of specific core symptoms was a response to longstanding criticism of AjD being difficult to distinguish from normal stress reactions as well as from clinical and subclinical presentations of other mental disorders (e.g., depression) (Bachem & Casey, 2018; Baumeister & Kufner, 2009; Casey et al., 2001). Even though AjD is the 7^th^ most used diagnosis in the mental health field (Reed et al., 2011), there are comparatively few empirical studies on AjD. Epidemiologic studies have found a high prevalence of AjD among people exposed to stressful experiences. For instance, Perkonigg et al. (2018) found a prevalence of 27.3% of AjD among people who lost their job. Moreover, a recent large-scale study among cancer patients also found a prevalence of 12.4% of AjD (Hund et al., 2016). An up to 12-fold increased risk of suicide emphasizes the high clinical relevance of AjD (Casey et al., 2015; Gradus et al., 2010). To advance the understanding of ICD-11 AjD, further research on its psychopathological nature and symptomatic characteristics is required (Bachem & Casey, 2018; Eberle & Maercker, 2022). As core features of AjD, the newly introduced preoccupation and failure to adapt symptoms are particularly relevant for future research.

The ICD-11 notes that preoccupation with the stressor or its consequences includes different cognitive phenomena, such as excessive worry, recurrent and distressing thoughts about the stressor, or constant rumination about its implications. Similarly, in past studies, preoccupation has often been defined using other cognitive symptoms, such as rumination or worry (e.g., Lehtonen et al., 2009). However, it is unclear how these different forms of repetitive thinking symptoms are differentiated and if preoccupation may not possess its own and independent symptom structure. In a recent review of the literature, a new characterization of preoccupation was proposed, in which preoccupation was defined as stressor-related factual thinking, which is time-consuming and often associated with negative emotions (Eberle & Maercker, 2022). This definition differentiates preoccupation from rumination, defined as negative and dysfunctional thinking, and worry, defined as anxiety-based and exclusively future-oriented thinking (Eberle & Maercker, 2022). Such a specific definition and distinction of major cognitive symptoms, including preoccupation, rumination, and worry, is highly needed in light of the numerous overlapping and conflicting constructs in the area of cognitive symptoms (for an overview, see Smith & Alloy, 2009). However, the validity of the proposed preoccupation characterization is unclear. Considering the current lack of research in this field, preoccupation needs to be further investigated in empirical studies, which could have fundamental implications for the understanding of cognitive symptoms in clinical psychology.

Similar to preoccupation, the current characterization of failure to adapt in ICD-11 AjD is rather rudimentary: The ICD-11 defines failure to adapt without providing details on possible psychopathological manifestations of this symptom cluster. Measurement instruments, such as the Adjustment Disorder New Module (ADNM; Einsle et al., 2010) or the International Adjustment Disorder Questionnaire (IADQ; Shevlin et al., 2020) describe failure to adapt symptoms as concentration problems, sleep disturbances or difficulties to achieve a state of inner peace. Previous research has drawn attention to the fact that failure to adapt seems to be a much more heterogeneous symptom cluster than propccupations (Bachem & Maercker, 2016; Levin et al., 2021). However, to our knowledge, no study has systematically explored other psychological problems of AjD patients that potentially fall under the category of failure to adapt symptoms. For example, individuals experiencing failure to adapt might also report problems such as memory issues or excessive fatigue. Identifying and incorporating such psychological problems into diagnostic processes could contribute to improving the clinical practices of AjD treatment.

Moreover, there is a possibility that failure to adapt is closely related to preoccupation processes, an assumption supported by studies showing a close association between concentration problems and repetitive thinking (e.g., Watkins & Roberts, 2020). There is also a chance that sleep disturbances are a consequence of increased repetitive thoughts about the distressing life event(s) (Takano et al., 2012), which could mean that failure to adapt and preoccupation are strongly interrelated processes. Insights in this area have the potential to discover major dynamics in AjD psychopathology and recovery. Empirical research that further explores the manifestations of failure to adapt in AjD patients is therefore essential.

Despite the ICD-11’s efforts to specify the clinical picture of AjD, the core symptom groups of preoccupation and failure to adapt should be further defined and differentiated. Shedding light on the psychopathological characteristics of these symptoms would likely improve the validity of AjD in research and clinical practice as previous problems related to AjD are essentially caused by the vague conceptual characteristics of this disorder. A qualitative bottom-up approach investigating different clinical presentations of AjD might provide useful results that could enrich past findings from quantitative analyses. The present study recognizes this potential and aimed to explore the characteristics and determinants of preoccupation and failure to adapt in qualitative interviews. For this purpose, a sample of individuals from an inpatient setting who suffered from AjD was investigated.

## Method

### Participants and Procedure

Participants were recruited within the University Hospital of Tours. They were first assed by regular psychiatrists who performed the diagnostic evaluation based on the ICD-11 criteria. Patients suffering from AjD received information about the current study and were invited to pariticipate. The diagnosticians knew that the study aimed to develop the understanding of ICD-11 AjD through a qualitative analysis, but this did not influence the section of the patient since all AjD patients were offered to participate. The interviews were conducted by the first author, who is a psychologist. Participants signed a consent form after receiving written and verbal information about the study. Next, they answered demographic questions about their age, sex, years of study after high school, and use of medication. Finally, they completed questionnaires assessing symptoms of AjD, anxiety and depression. Semi-structured interviews were then conducted to identify the determinants and characteristics of preoccupations and failure to adapt. These interviews were recorded with the participants’ consent, to enable qualitative analysis. They were then transcribed by the first two authors. All participants completed the study. The study was approved by the ethics committee of the University of Tours.

Sixteen participants (eight women) (mean age = 41.75 ± 18.28) were recruited during psychiatric consultations in a public hospital between May and October 2022. One interview had a duration of only 5 minutes because the patient presented an intellectual limitation. Otherwise, the duration of the interviews was between 12 and 55 minutes. Participants were at least 18 years old and had been diagnosed with AjD in a clinical interview by their regular psychiatrist, using the ICD-11 criteria. One patient also met the criteria for schizophrenia and another one for bipolar disorder. However, they had been well stabilized with medication and the emotional response they presented was clearly related to the event they experienced (a break-up) rather than their chronic mental disorder. Moreover, these two patients had received the diagnosis of schizophrenia or bipolar disorder during prior visits, but they did not present with psychotic, depressive or manic symptoms during the current visit. Such symptoms were absent for a long time due to well-balanced medication. Four participants had not completed high school, five participants completed high school and seven participants completed a college degree. Six patients received psychiatric medication at the time of the interview. Three patients received an antidepressant treatment, one a hypnotic treatment, three an antipsychotic treatment and one a benzodiazepine treatment. The mean scores and standard deviation of the different scales were 59.12 ± 11.06 (ADNM), 9.81 ± 4.82 (HAD-anxiety) and 10.59 ± 3.50 (HAD-depression). The stressful events they experienced are displayed in Table 1.

**Table 1:** Exposure to Critical Life Events

Dimension	Events mentioned (*n*)	Main event (*n*)
Break-up/divorce	5	3
Familial conflict	3	1
Conflict at work	2	0
Disease of a loved one	6	2
Death of a loved one	4	3
Jobless	3	0
Too much/too little work	2	0
Time pressure	4	0
New home	2	0
Financial problems	2	0
Own disease	6	5
Accident	2	0
End of a leisure activity	5	0
Quarantine due to an outbreak	2	0
Other event	5	2

### Measures

#### The Adjustment Disorder New Module (ADNM)

The ADNM-20 (Einsle et al., 2010) consists of two parts. In the first part, participants indicate stressful events that occurred during the past two years and have burdened them during the last six months. Then, participants indicate the most burdensome event(s), henceforth referred to as main events. Finally, they provide a symptom rating of ICD-11 core symptoms and accessory symptoms related to these events on a four-point Likert scale from 1 (never) to 4 (often). Previous results have found excellent psychometric properties within a French population (α = .92) (Vancappel et al., 2021). We also found good reliability in the present sample (α = .84). A score above 47 indicates the probable presence of AjD (Lorenz et al., 2016).

#### The Hospital Anxiety Depression Scale (HAD)

The HAD is a self-report questionnaire that assesses depression and anxiety (Zigmond & Snaith, 1983). Seven questions are related to anxiety and seven are related to depression. Participants answer multiple choice questions, withfour response options. The French version showed good psychometric properties (Cronbach alpha from .67 to .90) (Razavi et al., 1989). A score above seven indicates a borderline abnormal case and a score above 10 indicates the probable presence of depression or anxiety disorder.

### Semi-Structured Interview

A semi-structured interview schedule was conducted. The interview was developed based on the ICD-11 criteria for AD and the available questionnaires that assess AjD. It also left enough flexibility for the participants to mention content that was not already identified in classifications or questionnaires. The questions are presented below.

**Preoccupations**
- What do you think about the event?
- What is the content of your preoccupations?
- What do you feel when you are preoccupied with the event?
- What triggers preoccupations?
- How do the preoccupations stop?
- What do you do to stop your preoccupations?
- What are the consequences of your preoccupations?
- What do you feel about the event?

**Failure to adapt**
- How do you adapt to the event?
- How does the event impact your ability to relax?
- How does the event impact your ability to achieve inner peace?
- How did your expectations of the future change after you experienced the stressful event?
- How did your ability to work or carry out the necessary tasks in everyday life change after the event?
- What is the impact of the event on your daily life (in work, social relationship and leisure activities)?

The clinician explored the patient’s response to each question and asked if they had anything to add before moving on to the next question.

### Thematic Analysis

We used thematic analysis to process the data (Braun & Clarke, 2006). The interviews were transcribed and were first read for overall familiarization and then read again and coded using a double-coding procedure. The data were coded first by the first author and then by the second author; minor disagreements were resolved, and the codes were categorized into themes and sub-themes.

## Results

### Thematic Analysis

Six themes were identified. The number and percentage of participants who mentioned each theme and sub-theme are presented in Table 2.

**Table 2:** Number and Percentage of Participants Who Mentioned Each Theme and Sub-Theme

Theme / Sub-theme	*n*	%
Preoccupations triggers	16	100.0
Constance-uncontrollability	10	62.5
Reminders	10	62.5
Preoccupations and negative emotions	16	100.0
Anger-injustice	10	62.5
Anxiety-stress-worries	11	68.8
Frustration	1	6.3
Sadness	13	81.3
Guilt	10	62.5
Remorse-regrets	3	18.8
Powerlessness	8	50.0
Fear	10	62.5
Other	12	75.0
Strategies to stop preoccupations	16	100.0
Substances	3	18.8
Keeping the mind busy	13	81.3
Inability to set strategies	8	50.0
Suppression strategies	3	18.8
Consequences of preoccupations	10	62.5
Inner peace	6	37.5
Inability to relax	4	25.0
Envy	5	31.3
Sleep	3	18.8
Food intake	2	12.5
Manifestation of failure/difficulties to adapt	16	100.0
Ability to relax	13	81.3
Inner peace	6	37.5
Projections into the future	14	87.5
Dependence	1	6.3
Sense of utility	2	12.5
Efficacy	12	75.0
Others’ look	4	25.0
Sleep	1	6.3
Thoughts	16	100.0
Self-confidence	1	6.3
Motivation	11	68.8
Life	11	68.8
Social relationships	4	25.0
Injunction of adaptation	4	25.0
Difficulties of acceptance	3	18.8
Ruminations-impact of event	10	62.5
Strategies to address difficulties/failure to adapt	12	75.0
Adjustment strategies	8	50.0
Adjustment abilities	8	50.0
Resilience	1	6.3

### Theme 1: Preoccupation Triggers

The participants described what triggers their preoccupation. They mostly mentioned that their preoccupation “never stops” and that they have the event “always in mind”. They also mentioned that preoccupation is more frequent when their mind is free and not distracted by another task and when there is a reminder of the event (e.g., a message from the ex-partner, seeing the scar of a surgery, or a picture of a lost loved one). One patient who had experienced a break-up explained that he wakes up, looks for his partner in the bed and starts thinking about the event for the rest of the day.

### Theme 2: Preoccupations and Negative Emotions

All participants mentioned the presence of preoccupation and negative emotions. From the patients’ perspective, preoccupation and negative emotions were strongly interrelated. They described anger, explaining that “what happened is unfair”. One patient suffering from a somatic disease explained that she did not do anything to deserve her disease and that there is no justice. The patients mentioned anxiety and wondered a lot about what the event may cause in the future. For example, a patient who was engaged in an unfair lawsuit wondered what people will think about him after this event. They also referred to sadness, mostly explaining that life will not be the same for them. Many patients described inappropriate guilt, perceiving that the event was her/his fault. They also described powerlessness and fear. One patient who lost custody of her children said again and again “whatever I will do the judge will not give me my children back.”

### Theme 3: Strategies to Stop Preoccupation

Almost all participants mentioned different strategies aimed at stopping preoccupations. They frequently used substances (e.g., “I sometimes drink a bit of alcohol, but it makes my mood worse”), distraction strategies (e.g., “I keep my mind busy, do some shopping or read a bit”) and suppression strategies. For instance, a patient who lost a friend explained that he tried to bury his emotions about the event. Several patients also mentioned that they were not able to stop their preoccupation despite such efforts. When the patients were asked how the preoccupation stops, some of them responded “It never stops.”

### Theme 4: Consequences of Preoccupation

Interestingly, when asked to describe the negative consequences of preoccupation, participants mentioned several symptoms corresponding to the ICD-11 core symptom cluster of failure to adapt. They confirmed experiencing an impaired inner peace or ability to relax (“e.g., my thoughts are like in a circle and I cannot find inner peace”). They also described that preoccupations alter the quality of their sleep, their motivation for proper alimentation and their general level of energy. One patient explained that after his break-up he has “eaten nothing but surimi for weeks”.

### Theme 5: Manifestation of Failure/Difficulties to Adapt

Similarly, when patients described the negative consequences of the event more generally, further difficulties or failed attempts to adapt were mentioned. Mostly, the patients tended to describe failure to adapt as the direct consequences of the event and did not perceive how their attitude could be involved in their difficulties. They sometimes mentioned “it is not possible to adapt” to the event. Among the consequences, the patients described a disrupted inner peace or ability to relax (e.g., “I do not have inner peace”). They mentioned a negative impact of the event on their prospects for the future (e.g., “I do not picture myself in the future anymore”). They talked about a dependence on other people. For instance, one patient who suffered from a neurologic disorder that restrained her mobility said “I have gone from hyperactive to being a vegetable”. The patients also mentioned a lack of utility or efficacy and the related feeling that people may judge them (e.g., “People do not like someone who is complaining all the time”), a decrease of self-confidence and motivation (e.g., “I do not have energy anymore”). They mentioned a negative impact on social life or life more globally and an inability to accept what happened that disrupts daily life.

### Theme 6: Strategies to Address Difficulties/Failure to Adapt

Finally, the patients mentioned individual adaptation strategies. One patient who suffered from a break-up explained that he was telling himself that other people have also suffered from a break and tried to tell himself that things are going to be better. Patients developed abilities to cope with the situation. One patient who lost his wife explained that he changed his habits and that he kept doing things (e.g., going for walks, seeing friends) without his wife. Finally, some patients described experiences of growth. For instance, a patient who lost a close friend explained that this event made him stronger and that he enjoys more deeply the time spent with close people because of this event.

## Discussion

This study aimed to identify the characteristics and the determinants of preoccupations and failure to adapt among patients suffering from ICD-11 AjD. Overall, ample examples of ICD-11 core symptoms of preoccupations and failure to adapt were identified in patient reports, which confirms the validity of the ICD-11 AjD concept. More specifically, we found six themes in our analysis to describe the nature and context of the core symptoms: 1) Preoccupation triggers, 2) Preoccupations and negative emotions, 3) Strategies to stop the preoccupations, 4) Consequences of preoccupations, 5) Manifestation of failure/difficulties to adapt and 6) Strategies to address difficulties/failure to adapt.

Eberle and Maercker (2022) suggested a narrower definition of preoccupations than the one currently presented in the ICD-11, describing them as stressor-related factual thinking, which is time-consuming and associated with negative emotions. In line with this suggestion, the present study found that preoccupations were stressor-related and associated with negative emotions. They were also found to be time-consuming as patients reported thinking about the event all the time. However, our data show limited support for the suggestion that repetitive negative cognitions in AjD solely refer to factual thinking. The patients reported neutral, negative, factual and nonfactual thoughts related to the index stressors. The content of the preoccupations was broad and related to multiple topics: thoughts about responsibility, questions about the future or regrets about the past. In this way, some thoughts could be labeled as ruminations, worries, preoccupations or negative automatic thoughts, according to the different theoretical backgrounds of cognitive symptoms. Future research should undertake a more detailed examination of the different cognitive phenomena to determine if preoccupation in the narrower sense as suggested by Eberle and Maercker (2022) is the core characteristic of AjD or whether different kinds of cognitive phenomena are relevant to represent patients’ suffering. A combination of different cognitive symptoms, as it was found in the present study, also appears in other disorders, such as depression or generalized anxiety disorder (Muris et al., 2005; Smith & Alloy, 2009). However, some types of repetitive thoughts may be particularly prevalent in AjD. For example, it was shown that altough generalized anxiety disorder is characterized by both worry and rumination, worry has a more significant impact on its clincal presentation (Yang et al., 2014). Likewise, it is possible that while repetitive thoughts about a stressful life event in AjD might manifest as preoccupation, rumination, and worry, one of these symptoms could be particularly relevant in the psychopathological presentation. Clarifying the significance of such symptoms could enhance the clinical psychological classification of AjD.

Manifestations of failure to adapt symptoms included the difficulties mentioned in current AjD questionnaires (e.g. sleep problems, inability to find inner peace, decreased motivation) (Einsle et al., 2010; Shevlin et al., 2020), but also included additional experiences, such as disturbances in appetite, lowered sense of utility, and impaired social relationships. This finding raises the question of whether failure to adapt symptoms are adequately covered in existing AjD questionnaires. Additional research is needed to explore the diverse manifestations of failure to adapt and to determine which manifestations of failure to adapt may be most central for AjD patients. Here, a starting point could be to explore the concept of lack of recuperative ability (Maercker, 2017) as the core of this symptom group (e.g., sleep disorders, concentration disorders, inability to find inner peace, lowered sense of utility).

Concerning the pathogenesis of AjD, patient reports suggest that preoccupation may be the starting point of their difficulties and eventually result in failure to adapt. Specifically, manifestations of failure to adapt, such as difficulties finding inner peace or falling asleep, were described as a consequence of prolongued constant preoccupation with the stressor. This is in line with recent findings based on network analyses, which found that after a critical life event, preoccupation symptoms were most central in non-clinical samples whereas among participants with a suspected diagnosis of AjD, failue to adapt and functional impairment were most central (Levin et al., 2021, 2022). The present study strengthens the assumption that preoccupation plays an important role in the pathogenesis of AjD.

This study has several limitations. Due to the qualitative design, the number of participants was limited, making it difficult to generalize the conclusions. The research was also conducted in a single hospital, limiting the representativeness of the general population. The sample was also diverse with regard to the stressors experienced, age, medication. However, such diversity is representative of the patient group suffering from AjD. Moreover, a few patients had comorbid mental disorders. Even though their psychopathological state was clearly dominated by the current AjD symptoms, the additional disorders may have influced their cognitions and emotions, which were investigated in the present study. Finally, the use of thematic analysis is *per se* subjective. This means that the interpretation of the data may have been biased by the previous knowledge of the researchers.

Nevertheless, the present study highlighted the significance of stressor-related and emotionally aversive cognitions in AjD, which has clinical implications. Preoccupations should be a prime target in interventions as they seem to be crucial in the stress response and as they are related to maladaptive coping strategies such as alcohol consumption. A reduction in preoccupation symptoms during the earlier stages of the stress-response may be related to a decrease in failure to adapt symptoms. This assumption is consistent with interventions focused on other cognitive symptoms. For instance, it was found that worry causes mental impairment beyond the cognitive level (e.g., problem solving) and that in turn, a reduction of worry might reduce a broad range of psychological problems (Llera & Newman, 2020). Psychoeducation about the nature and maladaptive effects of preoccupation and cognitive restructuring or cognitive defusion (Assaz et al., 2023) could be used to address distressing repetitive thoughts.

## Data Availability

The dataset gathered and/or analyzed during the current study is available from the corresponding author on reasonable request.
